# Relationships between the fecundity of bark beetles and the presence of antagonists

**DOI:** 10.1038/s41598-022-11630-w

**Published:** 2022-05-09

**Authors:** Karolina Resnerová, Jolana Schovánková, Jakub Horák, Jaroslav Holuša

**Affiliations:** Department of Forest Protection and Entomology, Faculty of Forestry and Wood Sciences, Czech University of Life Sciences, Prague, Czech Republic

**Keywords:** Parasitology, Pathogens, Entomology

## Abstract

Although previous research has documented the occurrence of antagonists of bark beetles, the studies have only evaluated individual antagonists and have not assessed the overall effect of all antagonists on adult beetles. In this study, we determined which body-cavity antagonists were associated with a reduction in the fecundity and maternal gallery lengths of two important species of bark beetles: *Ips typographus* on Norway spruce and *I. cembrae* on European larch. We evaluated these relationships under natural conditions by collecting maternal females in galleries and examining their internal organs. The antagonists in the *I. typographus* hemolymph had significant negative associations with fecundity and gallery length. These antagonists were mainly nematodes and parasitoids in the hemocoel. In contrast, a positive association between gregarine presence and *I. typographus* fecundity was found. No antagonist that was likely to significantly alter *I. cembrae* fecundity or maternal gallery length was proven. Our study provides the first comprehensive assessment of antagonists that may have the potential impact on reduction the fecundity and thereby mass occurrence of these bark beetles.

## Introduction

The spruce bark beetle, *Ips typographus* (Linnaeus, 1758), is currently the most important pest of Norway spruce across Eurasia; the beetle is especially damaging to spruce stands that were planted on the warm side of the tree’s natural climatic range and that are now experiencing higher temperatures associated with climate change^1^. *I. typographus* has caused considerable damage to the entire range of spruce in recent years due to its eruptive outbreaks; it has contributed to the observed doubling in canopy mortality of spruce in Europe^2–4^. The large larch bark beetle *Ips cembrae* (Heer, 1836) has been reported as an important European larch pest in several European countries^5^. Adult *I. cembrae* attack weakened or healthy larches of all ages and at a wide range of altitudes^5–8^. However, in contrast to the mass occurrence of *I. typographus*, outbreaks of *Ips cembrae*, are rare and occur only locally on larches^5–7,9,10^. For example, damage by *I. cembrae* was reported for > 23,000 ha of young larches in Poland at the end of the 1990s^7^, and short-term outbreaks were triggered in central Europe by extreme drought in 2003^6,11,12^.

Fecundity is the physiological maximum potential reproductive output of an individual^13^- for bark beetles, fecundity refers to the number of eggs deposited by one female in its maternal gallery. The number of eggs laid by *Ips* bark beetles and the length of their maternal galleries are influenced by a number of factors, including the ambient temperature during oviposition^14,15^ and the population density of bark beetles on the infested tree^16–19^.

Both *I. typographus* and *I. cembrae* are polygamous species that use aggregation pheromones. Each male copulates multiple times with two or three females in the case of *I. typographus*^20^, and with two to five females in the case of *I. cembrae*^21^. The adult beetles mate in a nuptial chamber, and females of both species extend the nuptial chamber into a maternal gallery system (one system per female) in the phloem of the host tree^20,21^. Females of both species compete during the creation of gallery systems, and their larvae also compete, because the tunnels of the gallery system form in a two-dimensional space. Females deposit a single egg at regular intervals (1–2 eggs per day) along both sides of the maternal gallery but preferably on the side that least interferes with other gallery systems^22^. Each *I. typographus* female deposits up to 80 eggs^23^, and each *I. cembrae* female deposits up to 50 eggs^21^.

From the perspective of both species, the optimal density of maternal galleries is about 0.5 per dm^2^ of bark^24^. If the density is higher, the total production of eggs and the length of maternal galleries per female decreases due to oviposition competition^16,17^. To reduce competition, females may leave the gallery system prematurely and deposit eggs elsewhere^16,25,26^.

Antagonists may also be a factor limiting bark beetle females’ fecundity. Natural enemies of bark beetle include insectivorous birds, predatory insects, parasitoids, pathogens, and parasites^27^. In this study, we focused on groups of antagonists living within the body cavity of bark beetles where they may potentially reduce egg production^28,29^. These antagonists include taxonomically diverse pathogens, nematode parasites, and parasitoids that may, in some cases, cause serious pathological and behavioral changes in their hosts^27,30,31^. Although antagonists of *I. typographus* are well known, their effects on beetle fecundity have most often been studied in the laboratory^32,33^. Natural enemies of *I. cembrae* have been infrequently studied^34–36^, and their impacts on *I. cembrae* abundance and fecundity are largely unknown. Both positive^37^ and negative effects^38–41^ of antagonists on swarming, gallery construction, adipose tissue size or egg viability have been reported in bark beetles. Nevertheless, a comprehensive analysis of the effects of all antagonists simultaneously present under natural conditions has never been presented. In the current study, we evaluated the effect of multiple antagonists on the fecundity of *I. typographus* and *I. cembrae* under natural conditions.

To evaluate this effect, we performed a field study involving the measurement of maternal tunnels and the counting of eggs. Subsequent laboratory analysis of antagonists compared the fertility of females that were infected or not infected with individual antagonists. This study had three aims:

1. to determine whether maternal gallery length is related to the number of eggs laid,
2. to determine which antagonists are associated with reduced fecundity and shortened maternal galleries for *I. typographus* on spruce and for *I. cembrae* on larch,
3. and to determine whether the effect of antagonists is positive or negative and which group or species of antagonists are associated with the effect.

We tested the hypotheses that some of the antagonists would be significantly associated with reduced fecundity and gallery length^28,40^ of *I. typographus* and *I. cembrae* females.

## Materials and methods

### Sampling sites

**Figure 1:**
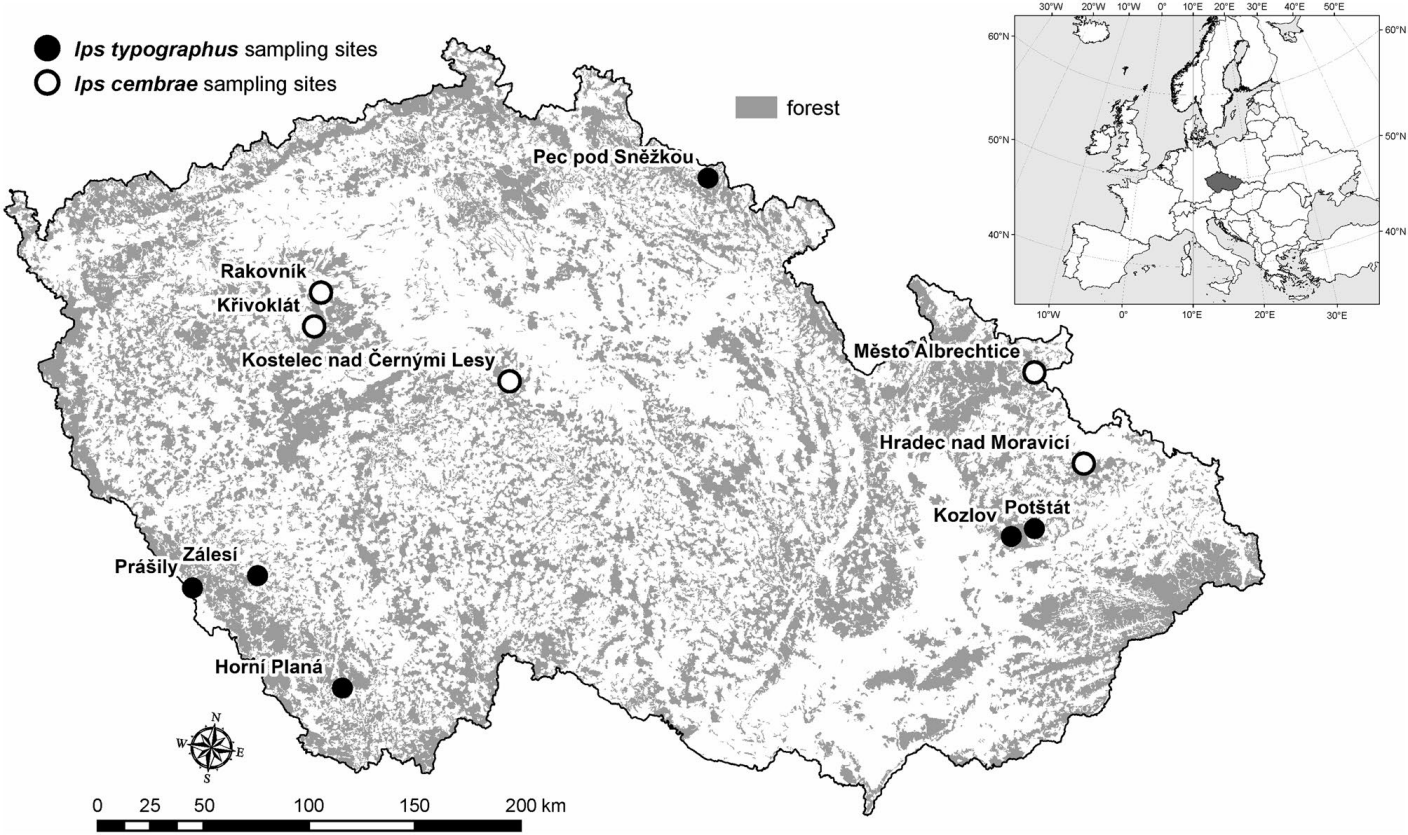
Sites in the Czech Republic where females of *I. typographus* and *I. cembrae* were collected in the period of 2009–2016. Sites are indicated by black circles for *I. typographus* and by white circles for *I. cembrae.* Software: ESRI 2020. ArcGIS Desktop: Release 10.8.1. Redlands, CA: Environmental Systems Research Institute.

*I. typographus* females were collected at six sites between 2009 and 2016 (Table 1), whereas *I. cembrae* females were collected at five sites between 2014 and 2015 (Table 1). The 11 sites were widely spread across the Czech Republic (Fig. 1) at elevations ranging from 264 to 790 m a.s.l. (Table 1). The six sites where *I. typographus* was sampled had “monocultures” of *Picea abies* (L.) H. Karst., and the five sites where *I. cembrae* was sampled had “monocultures” of *Larix decidua* Mill.; the sites included a maximum of 5 to 10% of other tree species (mostly larches, spruces, oaks, and pines). Sites were selected in stands exceeding 60 years and with a southern exposure. Only sites with bark beetle salvage cutting in previous years were selected.

**Table 1:** Background information on the 11 sites where *Ips typographus* and *I. cembrae* were sampled.

Bark beetle species	Sampling site	Year	Altitude (m a.s.l.)	GPS N Coordinates	GPS E Coordinates	Number of females (pcs.)
*I. typographus*	Horní Planá	2014, 2015	798	48.8300	14.2046	307
*I. typographus*	Kozlov	2010	620	49.6036	17.5357	85
*I. typographus*	Pec pod Sněžkou	2014, 2015,2016	796	50.6974	15.7506	565
*I. typographus*	Potštát	2009, 2011	502	49.6369	17.6517	232
*I. typographus*	Prášily	2014	883	49.0918	13.3708	215
*I. typographus*	Zálesí	2016	739	49.1651	13.6922	60
*I. cembrae*	Hradec nad Moravicí	2014	264	49.8710	17.8758	221
*I. cembrae*	Kostelec nad Č. L	2015	375	49.9329	14.8472	105
*I. cembrae*	Křivoklát	2015	425	50.0260	13.7953	57
*I. cembrae*	Město Albrechtice	2014	350	50.1629	17.5748	219
*I. cembrae*	Rakovník	2014	412	50.1421	13.8040	218

### Sampling design

The following sampling procedure was used at all sites:

In February, a series from 10 to 20 trap trees were prepared at each site. *Picea abies* were used as trap trees at sites with *I. typographus*, and *Larix decidua* were used as trap trees at sites with *I. cembrae*. Trees not infested by bark beetles with average parameters for the site (diameter from 25 to 35 cm, height from 20 to 35 m) were selected as trap trees and were felled. For Norway spruce trap trees, branches were removed from the felled trees and were placed on the trunks of the felled trees to reduce the drying of the phloem. For larch trap trees, branches were also removed from the felled trees but were not placed on the trunks.

Because the flight activity of the overwintering generation of both species begins in April^10,20^, the trap trees were monitored for the presence of bark beetle entry holes every week beginning in early April. The detailed inspection began after females initiated egg laying during May and June at all sites in all years of the study. Weekly monitoring included debarking of small areas (10 × 10 cm) for detecting the numbers and the life stages of bark beetles in the gallery systems.

When the population density of bark beetles on the trap trees ranged from 0.5 to 1.0 per 1 dm^2^, beetles were collected. The collection of the beetles always began when 2nd-instar larvae or later stages were present to ensure that the females had already completed egg deposition.

For beetle collection, knives were used to carefully remove the bark and expose the maternal galleries. If there was a female in the gallery, the length of the tunnel was measured with a ruler, and the eggs were counted. Only one maternal gallery from each gallery system was analysed to avoid overestimation of infection, because adults in one gallery system can transmit pathogens and parasites, resulting in a cluster of infected females. During debarking, the first complete maternal tunnel with a female present was selected.

Each female from measured galleries was placed in a 2-ml Eppendorf tube that was marked with the site, egg number, and gallery length.

Proportional random-stratified sampling^42^ was used when the number of plots (trap trees) randomly chosen at sites was proportional to the overall volume of bark beetle-infested wood in the previous year.

### Laboratory analysis

In the laboratory, the elytrae and wings were removed from each beetle, and the contents of its abdomen were dissected by gentle squeezing and were placed in a drop of distilled water.

All internal organs, i.e., intestine, adipose tissue, gonads, and hemolymph were examined with a Nikon Eclipse –Ci light microscope at 100–400 × magnification. If a male was found in the sample (based on the presence of male gonads), the beetle was excluded from the analysis.

The presence of nematodes, microsporidia, gregarines, viruses, and endoparasitoids based on visual inspection of internal organs was evaluated according as previously described^31,43,44^. Spores or cysts of pathogens and various developmental stages of parasites and parasitoids were mainly observed. Pathogens and parasitoids were identified to species. Nematodes were determined only to the group level according to the location in the host body. Accurate identification of nematodes would require molecular analysis, because only juvenile stages of nematodes are found in bark beetles. The only exception is *Contortylenchus* spp., which can be identified by the presence of females. Each collected female was assessed for the presence of antagonists but not for the abundance of antagonists.

### Statistical analysis

All analyses were done using R statistical software (v4.1.2; R Core Team 2021).

Because of the non-normal distribution of dependent variables (number of eggs and the lengths of galleries), a non-parametric procedure, the Spearman rank-order correlation coefficient, was used. The relationships between the dependent variables (number of eggs and the lengths of galleries) and the explanatory variables (intestinal nematodes, hemocoel nematodes, *Gregarina typographi*, *Mattesia schwenkei*, *Chytridiopsis typographi*, *ItEPV*, *Tomicobia seitneri, Contortylenchus* sp.) were determined using a generalized linear mixed-effect model (GLMM) with mgcv^45^ and nlme^46^ packages.

The possible collinearity of selected explanatory variables was verified using the HH package^47^ with VIF = 2 as a threshold.

Data for both dependent variables were analysed with a negative binomial distribution with a corresponding lambda value. Site was used as a random factor.

## Results

### Comparison of parameters reflecting the fecundity of *I. typographus* females

**Figure 2:**
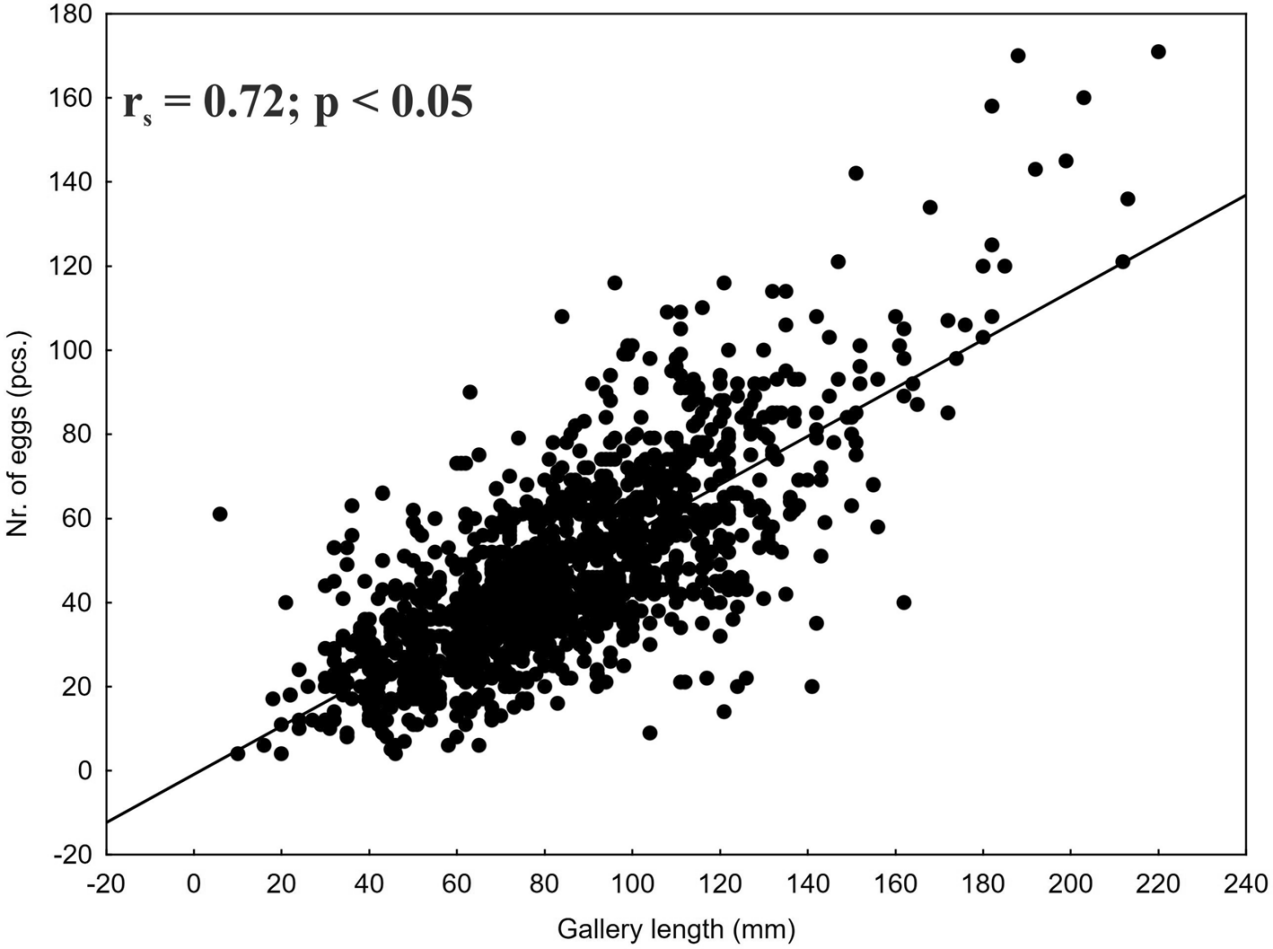
Scatterplot for the correlation between *I. typographus* maternal gallery length and the number of eggs per gallery.

A total of 1,464 *I. typographus* maternal galleries were analysed in this research. The average (± SE) length of the maternal galleries and number of eggs per maternal gallery was 83.2 mm (± 0.8) (min. = 6, max. = 220) and 47.0 (± 0.6) (min. = 4, max. = 171), respectively. Egg number per gallery was positively correlated with maternal gallery length (Fig. 2).

In total, seven groups of antagonists were detected in *I. typographus* females. Nematodes were found in the hemocoel of 21.2% of the females, which was the highest percentage of parasites detected. This was followed by intestinal nematodes, which were observed in 9.7% of the females. The only microsporidian identified during the study was *Chytridiopsis typographi* (Weiser 1954) Weiser 1970, which was found in 4.8% of the intestines of the females. A total of two apicomplexan species were identified in the samples: the neogregarine *Mattesia schwenkei* (Purrini, 1970) was found in 2.0% of the females, and the eugregarine *Gregarina typographi* (Fuchs, 1915) was found in 6.2% of the females. Viral infection by *Entomopoxvirus typographi* (*ItEPV*) (Weiser & Wegensteiner, 1994) was detected in 1.9% of the females. The only endoparasitoid species observed was *Tomicobia seitneri* (Ruschka, 1924), which was detected in 2.3% of the females (Table 2).

**Table 2:** Basic statistics (mean ± SE) of gallery pattern and number of *I. typographus* females in relation to presence or absence of antagonists of selected taxonomic groups.

Antagonist specification			No. of females [pcs.]		Length of maternal gallery (mm)		Number of cases	
Name	Taxonomy group	Location in the body of insect	Antagonists					
			Absence	Presence	Absence	Presence	Absence	Presence
Intestinal nematodes	Nematode	Intestine	47.1 ± 0.0.6	45.6 ± 1.7	83.2 ± 0.8	83.5 ± 2.2	1322	142
Hemocoel nematodes	Nematode	Hemolymph	47.7 ± 0.7	44.4 ± 1.1	83.8 ± 0.9	81.2 ± 1.6	1154	310
*G. typographi*	Gregarine	Intestine	46.3 ± 0.6	56.7 ± 2.1	82.4 ± 0.8	95.9 ± 2.9	1373	91
*M. schwenkei*	Neogregarine	Hemolymph	47.0 ± 0.6	44.4 ± 5.7	83.4 ± 0.8	73.1 ± 6.7	1435	29
*C. typographi*	Microsporidia	Intestine	47.0 ± 0.6	46.0 ± 2.0	83.2 ± 0.8	83.4 ± 2.5	1393	71
*ItEPV*	Virus	Intestine	47.2 ± 0.6	36.1 ± 3.7	83.5 ± 0.8	71.2 ± 4.7	1436	28
*Tomicobia seitneri*	Hymenoptera	Hemolymph	47.3 ± 0.6	33.5 ± 2.9	83.6 ± 0.8	68.6 ± 5.5	1431	33

The GLMMs indicated that the presence of *G. typographi* (Table 3) was positively related to the length of maternal galleries and to the number of eggs laid by *I. typographus* females, but that the presence of nematodes in the hemocoel was negatively related to the length of maternal galleries and the number of eggs laid by *I. typographus* females. Infestation by the endoparasitoid *T. seitneri* was negatively related to the number of eggs laid by females but was not related to the length of maternal galleries.

**Table 3:** Relationships between the number of eggs (pcs.) per *I. typographus* female or maternal gallery length (mm) and the presence of different groups of antagonists as indicated by GLMMs. Values in bold indicate a significant relationship at α = 0.05.

Independent variable	Variable	Value	SE	t	p
Number of eggs per female (pcs.)	Intercept	**3.8274**	**0.1066**	**35.8934**	** < 0.0001**
	Intestinal nematodes	− 0.0358	0.0394	− 0.9063	0.3649
	Hemocoel nematodes	− **0.1093**	**0.0306**	− **3.5807**	**0.0004**
	*Gregarina typographi*	**0.1618**	**0.0485**	**3.3334**	**0.0009**
	*Mattesia schwenkei*	0.0340	0.0816	0.4163	0.6773
	*Chytridiopsis typographi*	− 0.0276	0.0575	− 0.4806	0.6309
	*ItEPV*	− 0.1081	0.0830	− 1.3038	0.1925
	*Tomicobia seitneri*	− **0.1815**	**0.0770**	− **2.3567**	**0.0186**
Maternal gallery length (mm)	Intercept	**4.3967**	**0.0730**	**60.2280**	** < 0.0001**
	Intestinal nematodes	− 0.0112	0.0306	− 0.3678	0.7131
	Hemocoel nematodes	− **0.0721**	**0.0237**	− **3.0469**	**0.0024**
	*Gregarina typographi*	**0.1060**	**0.0376**	**2.8191**	**0.0049**
	*Mattesia schwenkei*	− 0.0911	0.0634	− 1.4364	0.1511
	*Chytridiopsis typographi*	0.0065	0.0446	0.1466	0.8835
	*ItEPV*	− 0.0692	0.0642	− 1.0780	0.2812
	*Tomicobia seitneri*	− 0.0938	0.0595	− 1.5758	0.1153

### Comparison of parameters reflecting the fecundity of* I. cembrae* females

**Figure 3:**
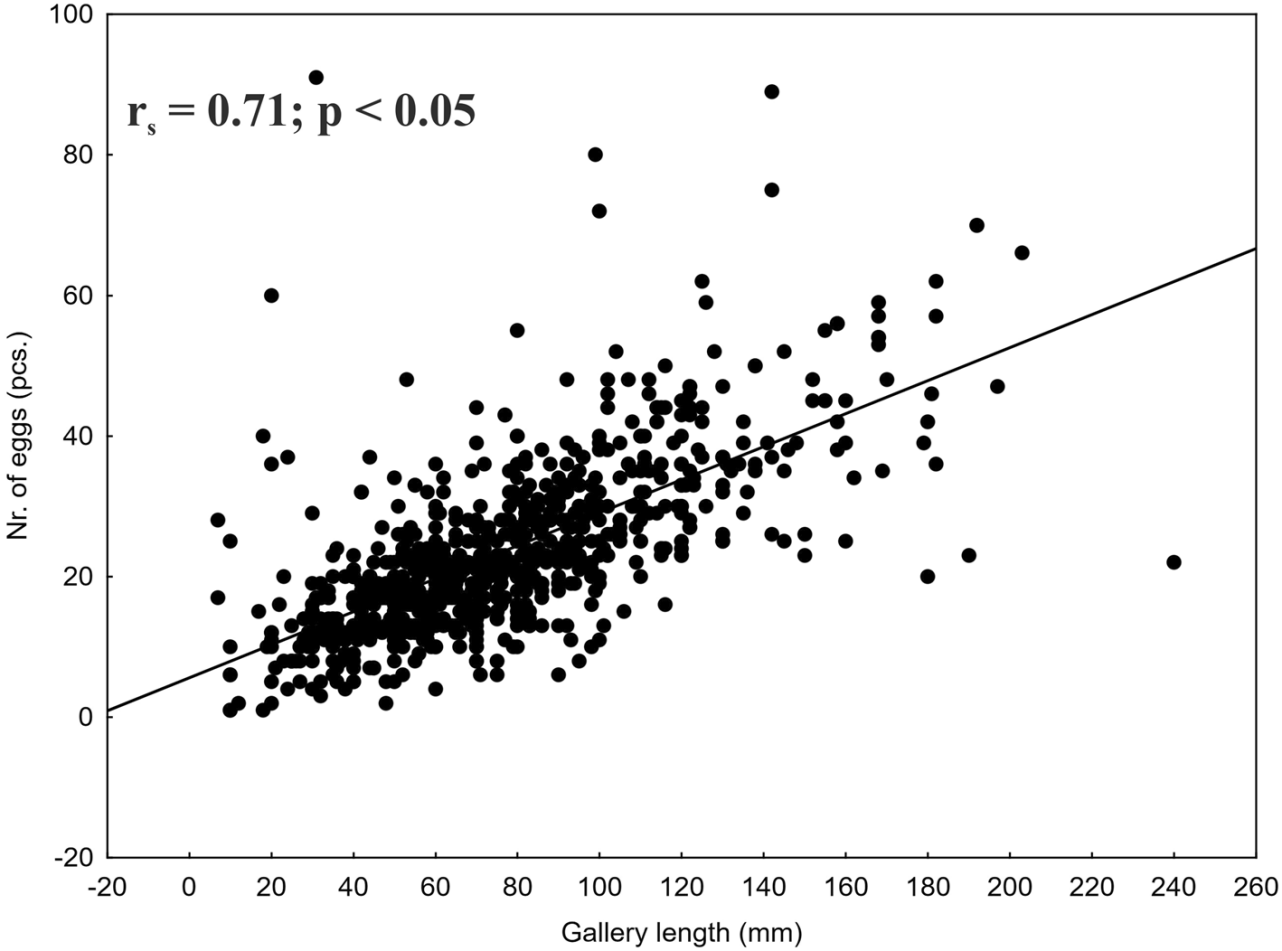
Scatterplot for the correlation between *I. cembrae* maternal gallery length and the number of eggs per gallery.

A total of 820 *I. cembrae* maternal galleries were found. The average (± SE) length of maternal galleries and number of eggs per maternal gallery was 76.0 mm (± 1.2) (min. = 7, max. = 240) and 23.5 (± 0.4) (min. = 7, max. = 91), respectively. As was the case with *I. typographus*, gallery length was positively correlated with the number of *I. cembrae* eggs per gallery (Fig. 3).

Four groups of antagonists were identified in *I. cembrae* females: intestinal nematodes (detected in 99.9% of the females), hemocoel nematodes (detected in 19.1% of the females), adult females of the nematode *Contortylenchus* sp. (detected in 1.6% of the females), and the microsporidian *Chytridiopsis typographi* (detected in 10.5% of the females) (Table 4).

**Table 4:** Basic statistics (mean ± SE) of gallery pattern and number of *I. cembrae* females in relation to presence or absence of antagonists of selected taxonomic groups.

Antagonist specification			No. of females [pcs.]		Length of maternal gallery (mm)		Number of cases	
Name	Taxonomy group	Location in the body of insect	Antagonists					
			Absence	Presence	Absence	Presence	Absence	Presence
Intestinal nematodes	Nematode	Intestine	13.0 ± 0.0	23.5 ± 0.4	50.0 ± 0.0	76.1 ± 1.2	1	819
Hemocoel nematodes	Nematode	Hemolymph	24.1 ± 0.5	20.8 ± 0.9	77.7 ± 1.4	68.9 ± 2.2	663	157
*Contortylenchus* sp.	Nematode	Hemolymph	23.5 ± 0.4	22.2 ± 2.2	76.1 ± 1.3	69.4 ± 5.8	807	13
*C. typographi*	Microsporidia	Intestine	24.0 ± 0.5	19.4 ± 1.1	76.5 ± 1.3	71.8 ± 3.7	734	86

The GLMM model indicated that the presence of any of the identified parasites and pathogens was not related to the length of *I. cembrae* galleries or with the number of eggs laid by *I. cembrae* females (Table 5).

**Table 5:** Relationships between the number of eggs (pcs.) per *I. cembrae* female or maternal gallery length (mm) and the presence of different groups of antagonists as indicated by GLMMs.

Independent variable	Variable	Value	SE	t	p
Number of eggs (pcs.) per female	Intercept	1.3953	1.3865	1.0064	0.3145
	Intestinal nematodes	0.3767	0.5008	0.7522	0.4521
	Hemocoel nematodes	− 0.0573	0.0453	− 1.2635	0.2067
	*Contortylenchus* sp.	0.1510	0.1413	1.0690	0.2854
	*Chytridiopsis typographi*	− 0.0638	0.0595	− 1.0712	0.2844
Maternal gallery length (mm)	Intercept	2.5153	1.4770	1.7029	0.0889
	Intestinal nematodes	0.2918	0.4483	0.6510	0.5152
	Hemocoel nematodes	− 0.0566	0.0413	− 1.3703	0.1709
	*Contortylenchus* sp.	0.0030	0.1289	0.0231	0.9815
	*Chytridiopsis typographi*	0.0638	0.0540	1.1803	0.2382

## Discussion

Several antagonists were associated with the number of deposited eggs and maternal gallery length of *I. typographus*. We found both positive and negative relationships between the presence of of “antagonists” and *I. typographus* fecundity. In particular, the number of *I. typographus* eggs and the length *I. typographus* maternal galleries were negatively related with presence of nematodes in the hemocoel, and the number of *I. typographus* eggs was negatively related with the presence of the endoparasitoid *T. seitneri* in the body cavity. Surprisingly, the presence of gregarines was positively related with the two variables, i.e., gregarines seemed to have increased *I. typographus* fecundity and maternal gallery length. This result suggests that, rather than being a bark beetle pathogen as is usually assumed, *G. typographi* may be more of a mutualist. The occurrence of other antagonists was not associated with the number of eggs laid or the gallery length per *I. typographus* female. In contrast to *I. typographus* fecundity, *I. cembrae* fecundity was not significantly related to the presence of antagonists, although means tended to be lower when nematodes were present in the hemocoel and when *Contortylenchus* sp. or *Chytridiopsis typographi* was detected. Both of the latter two hemocoel antagonists are significantly associated with reduced fecundity of *I. typographus* females under natural conditions. In the case of nematodes, however, we recommend further research focusing on the accurate detection of species that reduce bark beetle fecundity.

The mean number of eggs deposited by one *I. typographus* female was 47, and the mean length of the galleries was 83 mm. These values are consistent with previous reports^18,20,48^. The number of eggs laid in the absence of spatial competition for the first maternal gallery have been reported to range from 35 to 50^25^, which suggests that spatial competition was not a major factor affecting *I. typographus* females in the current study. For the less-studied *I. cembrae*, the mean length of the galleries was 76 mm, which is within the previously reported range^49^ but is lower than the range of 100 to 115 mm reported by^50^. The average number of eggs per *I. cembrae* female (n = 24) was in the range of previously reported values^21^. Because bark beetle females gradually bore the maternal galleries and lay eggs in them continuously, it is reasonable that gallery length is positively correlated with the number of eggs deposited for both species, as documented in the current study and in previous studies^16,18^. This close relationship between fecundity and gallery length suggests that it is a standard property for both species of bark beetles.

A total of four pathogens of *I. typographus* and one of *I. cembrae* were recorded. *Chytridiopsis typographi* microsporidia were found in the intestine of both species. It is a common pathogen reported in most bark beetles of the genus *Ips*^31^. The infection was found in the midgut epithelium in the form of cysts. Our results suggest that this microsporidian has little or no effect on the fecundity of the two species of bark beetles. The question, therefore, remains whether *C. typographi* is indeed a pathogen, because only two studies have assessed the effects of *C. typographi* on bark beetle populations. One laboratory study reported 100% mortality of infected bark beetles within 60 days^51^-the long period between infection and death, however, could allow females to complete gallery construction and egg deposition. A second study compared *C. typographi* infection rates in *I. typographus* collected in pheromone traps over a 10-year period; the results suggested that infection might reduce flight ability and interfere with pheromone perception^37^.

The neogregarine *Mattesia schwenkei* is the only pathogen thus far detected in the adipose tissue of bark beetles^35,52^. The infection level, however, is usually very low^48,53^, which is consistent with our findings. Although this pathogen breaks down the fat in the hemocoel and apparently reduced beetle fecundity and gallery length in the current study, the reductions were not statistically significant. *M. schwenkei* is thought to possibly reduce the establishment of a sister brood of bark beetles and to reduce the survival of overwintering bark beetles^54^. Similar effects have been assumed to result from infection of bark beetles by the *ItEPV* virus, which is usually found in the middle intestine^54^. In the current study, the virus was associated with a 23% reduction in the number of *I. typographus* eggs deposited and a 13% reduction of *I. typographus* gallery length, but the reductions were not statistically significant.

The only organism that was positively associated with increases in *I. typographus* fecundity and gallery length was the gregarine *Gregarina typographi*. Gregarines are thought to have many kinds of relationships with their hosts, including symbiotic, mutualistic, and parasitic relationships^55^. *G. typographi* has been found in many *Ips* species and is generally considered a pathogen of the anterior part of the intestine lumen^27,56–58^. Horizontal transmission of *G. typographi* occurred between beetles via spore ingestion in the nuptial chambers^57^. Evidence of a negative effect of gregarines on bark beetles is rather speculative. One report^37^ suggested a positive influence of gregarines on *I. typographus* flight, and *G. typographi*-infected beetles are captured more frequently in pheromone traps than uninfected beetles*.* This pathogen induces a specific within-year low mortality in beetle populations. For the first time, our results show a statistically significant positive association between the presence of gregarines and *I. typographus* fecundity and gallery length. The life cycles of gregarines differ significantly from those of most other apicomplexans because gregarines generally utilize only one group of host organisms^55^. The high degree of host specificity of gregarines^55^ suggests the coevolution of gregarines and their hosts^59^. We therefore suspect that gregarines are not parasites of bark beetles but instead are part of the host’s non-damaging natural flora. *G. typographi* might even contribute to *I. typographus* fitness via symbiosis. ^60^reported that the mechanical and physiological damage caused by gregarines to the midgut according^61^ can be easily repaired and that the pathological effects of gregarines are generally unimportant.

One adult bark beetle may contain a very large number of nematodes. It is not uncommon to find bark beetles with hundreds of nematode larvae in the gut, in the hemocoel, and under the elytrae^62^. Nematodes associated with bark beetles can be either commensal or parasitic. In commensal relationships, the effects on hosts are minimal, and host fitness is not reduced^38^. The influence of parasitic nematodes on the fecundity of bark beetles has been documented in several studies^63,64^. The nematodes that develop inside the beetle's body have been shown to remove nutrients from their host. This is evidenced by the reduced number of fat cells in infected bark beetles^40,65^ and by the reduced level of proteins in the hemolymph^39,66^.

*Parasitorhabditis* spp. and *Cryptaphelenchus* spp. are two dominant genera of nematodes found in bark beetle intestines^67^. Because the occurrence of intestinal nematodes in the two bark beetles of the current study was unrelated to the number of eggs laid or gallery length, we assume that these species do not reduce fecundity or gallery length. This is inconsistent with the findings of^68^, who found significant damage to gastrointestinal epithelial cells in *Ips sexdentatus* individuals parasitized by *Parasitorhabditis* spp. In a subsequent study^29^, reported a slight decrease in the density of oviposition incisions and in the number of eggs in *Ips sexdentatus* individuals parasitized by *Parasitorhabditis* sp. In the current study, the presence of nematodes in the intestines of *I. typographus* was associated with a 2-egg per female decrease in fecundity.

Three species of nematodes, *Contortylenchus diplogaster* (v. Linstow, 1890) Rühm 1956, *Parasitylenchus dispar* (Fuchs, 1915), and *Cryptaphelenchus* sp., were previously found in the hemocoel of *I. cembrae* and *I. typographus*^36,67^. It was revealed that the hemocoel of individual beetles can be simultaneously infected with multiple species of parasitic nematodes^38,67,69^. Although *Parasitylenchus* spp. may kill bark beetles under particular circumstances^70^, studies of *Contortylenchus* species have not demonstrated any lethal effects^30^. The fatal effects of nematodes on bark beetles could be easily overlooked in nature because infested dead beetles are difficult to detect but living and heavily infested beetles are easy to detect^27^.

In several studies in Europe, *Contortylenchus diplogaster* was the nematode most frequently found in the hemocoel of *I. typographus* and *I. cembrae*^34,36^. In the current study, the presence of nematodes in the hemocoel was associated with a significant decrease (7%) in *I. typographus* fecundity and a significant decrease (3%) in the length of *I. typographus* maternal galleries. These values are much lower than the 20 to 50% reduction in the number of eggs and the 25 to 27% reduction in gallery length reported by ^39^. Lieutier et al.^40^ reported that females of *Ips sexdentatus* parasitized by *Parasitaphelenchus* sp. and *Contortylenchus* sp. had smaller fat bodies and ovaries and less developed terminal oocytes than non-parasitized individuals. Such effects on fat bodies ovaries could delay the maturation of oocytes.

In our study, intestinal nematodes were found in all *I. cembrae* adults except one. We were therefore unable to determine the association between *I. cembrae* fecundity and the presence of intestinal nematodes. On the other hand, the presence of nematodes in the hemocoel rather than in the intestine was associated with a reduction in the fecundity of *I. typographus*. Perhaps this difference was due to differences in the proportion of beetles with parasitic nematodes in the hemocoel vs. intestine, i.e., nematodes were present in the intestines of nearly 100% of *I. cembrae* females. According to^36^, *C. diplogaster* is mainly found in the hemocoel of *I. cembrae* but can also occur in the intestine and malpighian tubules after being ingested with frass in the gallery^27^. With such a level of parasitism, the individual stages of nematodes could appear not only in the hemocoel but also in the intestines, making the clear identification of these groups difficult.

Two common obligate endoparasitoids were previously identified in *I. typographus* adults in Europe: *Tomicobia seitneri* and *Ropalophorus clavicornis* (Wesmael, 1835)^27,33,44^. In our study, we found only one pteromalid endoparasitoid, *T. seitneri*; the female of the latter endoparasitoid locates its host via a host aggregation pheromone^71,72^. Females oviposit directly through the thorax or elytra of bark beetle adults. In our study, we found both eggs and larvae of *T. seitneri* but in only 2% of analyzed females. The level of *T. seitneri* parasitism varied considerably in previous reports and ranged from 20 to 100% in^73^, from 0 to 19% in^44^, and from 0 to 35% in^73^; the level of parasitism apparently depends on the time of beetle collection and other factors. Our results are in line with the predictions of ^28^ because infected females continue to lay eggs, and the parasitoids do not hatch until after eggs are deposited. We found that the presence of *T. seitneri* was associated with an almost 16% reduction in *I. typographus* fecundity, but reductions as high as 30% were previously reported^28^, and even higher reductions were reported for *Tomicobia tibialis* Ashmead, 1904 on *Ips pini* females, i.e., parasitized *I. pini* females produced 50% fewer offspring than unparasitized females^41^. On the other hand, gallery length is not affected by *Tomicobia* parasitism, because infected females still create galleries. The parasitoid apparently destroys the eggs in the host's body and thereby prevents the eggs from being laid. *Tomicobia* larvae feed on the tissue in the hemocoel. The adult wasp gnaws its way out of the bark beetle elytral declivity and leaves the gallery^27^. *Tomicobia seitneri* has not been found in *I. cembrae* beetles and is not known in the literature as a parasitoid of *I. cembrae*^35^.

A number of known antagonists occurring in the adult stages were not detected in our research and deserve additional study. On the other hand, the prevalence of some pathogens is so low^27,31,74^ that we suspect that they are unlikely to influence the populations of the two bark beetles. Another question concerns the effects of multiple antagonists in one host, which has rarely been studied. With simultaneous infection of hosts by multiple antagonists, the negative effects on the host may increase in some cases but decrease in other cases^75,76^.

In conclusion, the current report provides the data on pathogens, nematodes, and endoparasitoids of two bark beetles, *I. typographus* and *I. cembrae* in natural conditions. This information is important because these bark beetles antagonists could potentially reduce beetle fecundity, which can in turn greatly affect the severity of outbreaks by these beetles. The results of our study should be included in models of forest landscape dynamics. In some forest landscape and disturbance model (e.g., iland^77^), a formula should be added to the mortality calculation, which at least partially accounts for the effects of pathogens and other biotic agents on pest mortality. The influence of individual pathogenic organisms on the fitness of beetles is often beyond the scope of the models. However, if the life cycles of biotic agents are sufficiently described and their pathogenicity is known, it should be possible to add this information into the mortality calculations in the models and thus make the models more accurate. Disturbance models are evolving very fast and becoming sufficiently modular so that they could be enriched by new research results.

## Data Availability

The datasets generated and/or analyzed during the current study are available from the corresponding author KR on reasonable request.
